# 3D Reconstruction and physical renal model to improve percutaneous punture during PNL

**DOI:** 10.1590/S1677-5538.IBJU.2018.0799

**Published:** 2019-12-17

**Authors:** Lorenzo Bianchi, Riccardo Schiavina, Umberto Barbaresi, Andrea Angiolini, Cristian V. Pultrone, Fabio Manferrari, Barbara Bortolani, Laura Cercenelli, Marco Borghesi, Francesco Chessa, Elisa Sessagesimi, Caterina Gaudiano, Emanuela Marcelli, Eugenio Brunocilla

**Affiliations:** 1 University of Bologna Department of Urology Bologna Department of Urology, University of Bologna, Bologna, Italy; 2 Department of Experimental, Diagnostic and Specialty Medicine (DIMES), Cardio-Nephro-Thoracic Sciences Doctorate, University of Bologna, Bologna, Italy; 3 Department of Experimental, Diagnostic and Specialty Medicine (DIMES), Laboratory of Bioengineering, University of Bologna, Bologna, Italy; 4 Department of Radiology, Sant'Orsola-Malpighi Hospital, University of Bologna, Bologna, Italy

## Abstract

**Introduction and Objectives:**

We aim to present the use of 3D digital and physical renal model (1–5) to guide the percutaneous access during percutaneous nephrolithotripsy (PNL).

**Materials and Methods:**

We present the clinical case of a 30 years old man with left renal stone (25x15 mm). A virtual 3D reconstruction of the anatomical model including the stone, the renal parenchyma, the urinary collecting system (UCS) and the skeletal landmarks (lumbar spine and ribs) was elaborated. Finally, a physical 3D model was created with a 3D printer including the renal parenchyma, UCS and the stone. The surgeon evaluated the 3D virtual reconstruction and manipulated the printed model before surgery to improve the anatomical knowledge and to facilitate the percutaneous access. In prone position, combining ultrasound and fluoroscopy implemented by the preoperative anatomical planning based on the 3D virtual and printed model, an easy and safe access of the inferior calyx was achieved. Then, the patient underwent PNL using a 30 Fr Amplatz sheet with semi-rigid nephroscope and ultrasound energy to achieve a complete lithotripsy of the pelvic stone.

**Results:**

The procedure was safely completed with 1 single percutaneous puncture (time of puncture 2 minutes). Overall surgical time was 90 min. No intra and postoperative complications were reported. The CT scan performed before discharge confirmed a complete stone free state.

**Conclusion:**

The 3D-guided approach to PNL facilitates the preoperative planning of the puncture with better knowledge of the renal anatomy and may be helpful to reduce operative time and improve the learning curve.

## ARTICLE INFO

**Available at: http://www.intbrazjurol.com.br/video-section/20180799Bianchi_et_al**

**Int Braz J Urol. 2019; 45 (Video #25): 1281-2**
